# AGEs promote atherosclerosis by increasing LDL transcytosis across endothelial cells via RAGE/NF-κB/Caveolin-1 pathway

**DOI:** 10.1186/s10020-023-00715-5

**Published:** 2023-08-21

**Authors:** Meng Shu, Wenzhuo Cheng, Xiong Jia, Xiangli Bai, Ying Zhao, Yajing Lu, Lin Zhu, Yan Zhu, Li Wang, Yan Shu, Yi Song, Si Jin

**Affiliations:** 1 Present Address: Department of Endocrinology, Institute of Geriatric Medicine, Liyuan Hospital, Tongji Medical College, Huazhong University of Science and Technology, 39 Lake Road, East Lake Ecological Scenic, Wuhan, 430077 Hubei China; 2 Department of Pediatrics, Tongji Hospital, Huazhong University of Science and Technology, Wuhan, Hubei China

**Keywords:** AGEs, RAGE, Diabetic atherosclerosis, Caveolin-1, LDL transcytosis

## Abstract

**Objective:**

To elucidate the mechanism whereby advanced glycation end products (AGEs) accelerate atherosclerosis (AS) and to explore novel therapeutic strategies for atherosclerotic cardiovascular disease.

**Methods and results:**

The effect of AGEs on low-density lipoprotein (LDL) transcytosis across endothelial cells (ECs) was assessed using an in vitro model of LDL transcytosis. We observed that AGEs activated the receptor for advanced glycation end products (RAGE) on the surface of ECs and consequently upregulated Caveolin-1, which in turn increased caveolae-mediated LDL transcytosis and accelerated AS progression. Our molecular assessment revealed that AGEs activate the RAGE-NF-κB signaling, which then recruits the NF-κB subunit p65 to the RAGE promoter and consequently enhances RAGE transcription, thereby forming a positive feedback loop between the NF-κB signaling and RAGE expression. Increased NF-κB signaling ultimately upregulated Caveolin-1, promoting LDL transcytosis, and inhibition of RAGE suppressed AGE-induced LDL transcytosis. In *ApoE*^−/−^ mice on a high-fat diet, atherosclerotic plaque formation was accelerated by AGEs but suppressed by EC-specific knockdown of RAGE.

**Conclusion:**

AGEs accelerate the development of diabetes-related AS by increasing the LDL transcytosis in ECs through the activation of the RAGE/NF-κB/Caveolin-1 axis, which may be targeted to prevent or treat diabetic macrovascular complications.

## Introduction

Diabetes mellitus (DM) is a significant risk factor for atherosclerotic cardiovascular disease (ASCVD) (Strain and Paldánius 2018). DM patients have approximately 2–4 times greater risk of developing ASCVD than non-DM patients (Cheng and Kishore 2020; Sun et al. 2022). Currently, ASCVD is considered the primary cause of death in DM patients. Thus, it is vital to explore the pathogenesis of atherosclerosis (AS) in DM.

According to the lipid “response-to-retention hypothesis” in AS, apolipoprotein B (apoB)-containing lipoproteins, principally low-density lipoprotein (LDL), cross through endothelial cells (ECs) and deposits in the subendothelial matrix of the tunica intima of arteries as the key initiating step in atherogenesis (Tabas et al. 2007; Fogelstrand and Borén 2012). Transcellular transport of LDL is implemented by the movement of LDL particles across an intact endothelial barrier via a transport process called transcytosis (Muradashvili et al. 2014). Caveolae, flask‐shaped plasma membrane invaginations, play essential roles in regulating lipid homeostasis, membrane tension, trafficking, and clathrin‐independent endocytosis (Yu et al. 2017). Caveolae-mediated transcytosis is believed to be the primary mechanism of LDL permeability across the continuous EC barrier (Sun et al. 2010). We have recently confirmed that DM patients have higher levels of Caveolin-1, a major membrane-bound scaffolding protein of the caveolae, in their vascular endothelium than patients without DM, and that a high blood glucose level increases Caveolin-1-mediated LDL uptake by ECs and transcytosis across the endothelium (Frank et al. 2004; Bai et al. 2020a, 2020b). Therefore, Caveolin-1 is essential for LDL transcytosis (Yang et al. 2016; Ramírez et al. 2019).

Advanced glycation end products (AGEs) are the end products of non-enzymatic glycosylation of proteins, lipids, and nucleic acids with glucose and other reducing sugars (Singh et al. 2001). Under normal circumstances, the levels of AGEs in the body gradually increase with age and blood glucose levels (Miyata et al. 2000; Nowotny et al. 2015). The level of circulating AGEs in DM patients is correlated with carotid media thickness and deposition of AGEs in atherosclerotic plaques in DM patients was confirmed (Kaneda et al. 2002; Chilelli et al. 2013; Wang et al. 2018). DM-related microvascular disorders are caused by AGEs rather than hyperglycemia and ultimately increase the risk of macrovascular complications (Chilelli et al. 2013). These data suggest that AGEs retention can promote AS in DM patients. However, the specific role of AGEs in atherosclerotic plaque formation remains unclear.

RAGE is a multi-ligand receptor that belongs to the type-I immunoglobulin superfamily and is expressed on the surface of various cells, including fibroblasts, epithelial cells, and ECs (Ramasamy et al. 2011). RAGE is the main receptor of AGEs and cellular responses stimulated by AGEs are initiated upon activation of the AGE receptors on the cell surface (Harja et al. 2008). Upon the ligation with AGEs, RAGE can modulate intracellular signal transduction pathways, such as the NF-ĸB signaling pathways, activate pro-inflammatory responses and trigger a cascade of pro-atherogenic responses (Chaudhuri et al. 2018; Hudson and Lippman 2018). Moreover, AGEs can damage vascular ECs, promote the adhesion and aggregation of leukocytes, stimulate the proliferation of vascular smooth muscle cells, and promote the pathogenesis of AS (Nowotny et al. 2015; Byun et al. 2017). Additionally, it has been demonstrated that NF-ĸB upregulates Caveolin-1 expression to induce LDL transcytosis (Zhang et al. 2014), and we considered whether AGEs might also promote this process at high blood glucose level. Indeed, there is evidence that RAGE is localized to caveolae (Lisanti et al. 1994), and the available data demonstrate a strong link between AGEs and AS, which is most likely regulated through a signaling pathway mediated by RAGE. However, the underlying molecular mechanisms are still unknown.

In this study, we used a validated in vitro model of LDL transcytosis, whereby we observed that AGEs induced LDL transcytosis by promoting Caveolin-1 expression through the nuclear translocation of the transcription factor NF-ĸB as a result of a positive feedback loop between RAGE activity and the NF-ĸB signaling. Consequently, a pathophysiological cascade ensued. We also established a mouse model of AS by feeding *ApoE*^−/−^ mice a high-fat diet (HFD) (Bu et al. 2010; Zhao et al. 2022) and discovered that recombinant adeno-associated viral vector (rAAV)-mediated EC-specific RAGE knock-down suppressed the stimulatory effect of AGEs on AS in vivo. Our findings shed light on the pathogenesis of diabetic AS and present RAGE as a potential therapeutic target.

## Materials and methods

### Cell culture

Human umbilical vein endothelial cells (HUVECs) were obtained from Wuhan Union Hospital Regenerative Medicine Center, Hubei, China. HUVECs were routinely cultured in DMEM (SH30022.01, HyClone, USA) with 10% fetal bovine serum (2500, ScienCell, Carlsbad, CA, USA) and 1% penicillin/streptomycin (0503, ScienCell, Carlsbad, CA) at 37 °C in a humidified atmosphere of 5% CO_2_. The cells were sub-cultured (1: 3) at 80% confluence.

### Reagents and antibodies

BSA and AGE-conjugated BSA (AGEs-BSA) (#2221–10) were purchased from Biovision (Milpitas, CA, USA). LDL (YB-001) and DiI-conjugated LDL (DiI-LDL) (YB-007) were obtained from Yiyuan Biotechnology. FITC (46950) was purchased from Sigma-Aldrich. The antibodies against Caveolin-1 (3267, 1:1000) and GAPDH (8884, 1:1000) were acquired from Cell Signaling Technology (Beverly, MA, USA), antibody against p-p65 (AP0124, 1:500) was acquired from ABclonal (Wuhan, China), and those against RAGE (16346-1-AP, 1:2000), NF-κB p65 (10745-1-AP, 1:1000), and IκBα (10268-1-AP, 1:5000) were acquired from Proteintech (Wuhan, China). DAPI and Alexa-Fluor-488–tagged secondary antibodies were purchased from Beyotime (Shanghai, China). PDTC (HY-18738) was obtained from MedChem Express (Monmouth Junction, New Jersey, USA).

### Cell-viability assay

We determined the viability of HUVECs upon application of various concentrations of BSA or AGEs-BSA for various periods. First, HUVECs were seeded into 96-well plates (5 × 10^3^ cells with 100 µL culture medium per well) and cultured for 24 h. Subsequently, 10 µL of the cell counting kit-8 (CCK-8) solution was applied to each well per the manufacturer's instructions (Dojindo, Shanghai, China), followed by incubation of the cells for 4 h at 37 °C. Finally, the absorbance of the cells at 450 nm was quantitated using a microplate reader.

### LDL transcytosis

An in vitro model of LDL transcytosis was established as reported previously (Bian et al. 2014; Bai et al. 2020a, 2020b). In brief, HUVECs were seeded (2 × 10^5^ cells per insert) on polyester membranes in transwells (Costar, USA). The integrity of the cell monolayer was assessed using a previously described method (Cankova et al. 2011; Tanigaki et al. 2018). The non-competitive and competitive inserts were both assigned to the same group of cell monolayers with similar integrity. To quantitate the total trans-endothelial LDL content, the non-competitive inserts of cells were incubated with 50 μg/mL FITC-LDL. The para-cellular LDL transport was quantitated by incubating the competitive inserts of cells with 50 μg/mL FITC-LDL and 300 μg/mL unlabeled LDL. Uncoupled FITC was discarded using dialysis against PBS for 72 h at 4 °C after the samples were taken from the lower chambers. A fluorescence spectrophotometer (TECAN, INFINITE F200PRO) with an excitation wavelength of 490 nm and an emission wavelength of 520 nm was used to measure the signal intensity of the coupled FITC. The background fluorescence of the medium (Opti-MEM, Gibco) was measured and then subtracted from the calculated FITC fluorescence intensity to identify the net intensity per sample. LDL transcytosis was quantitated by subtracting the net FITC fluorescence intensity in the competitive insert from that in the non-competitive insert.

### Confocal imaging of HUVECs to analyze LDL uptake

HUVECs were pre-treated as indicated and then incubated with 50 μg/mL DiI-LDL for 24 h. Following a PBS wash, the cells were immediately fixed with 4% paraformaldehyde (PFA), and then the nuclei were stained with DAPI. A laser scanning confocal microscope (FV3000; Olympus) with a 60 × objective lens was used to photograph the stained cells. To evaluate fluorescence intensity, three separate fields of view were collected from each biological replicate. For all samples within each experiment, user-defined fluorescence intensity thresholds were set and applied consistently. Throughout the quantification, the researcher was blinded to the treatment group. The fluorescence intensities of the confocal images were calculated and then normalized to the number of viable cells by using the Image J software.

### Flow cytometry

HUVECs were grown to confluence in 12-well plates. The LDL uptake assay was initiated by incubating the cells for 3 h in serum-free Opti-MEM (Gibco) supplemented with 50 μg/mL Dil-LDL. The cells were then washed twice with PBS and detached using trypsin (without EDTA). The cellular Dil-LDL uptake was quantitated by subtracting the background fluorescence from the fluorescence of the Dil-LDL–treated samples by using flow cytometry (Mindray, China) and expressed as mean fluorescence intensity (MFI). FSC/FSS scatter diagrams based on a total of 10,000 events were plotted.

### Small interfering RNA (siRNA) transfection

HUVECs were cultured to 80% confluence in 6-well plates. The cells were then transiently transfected with an siRNA targeting RAGE or Caveolin-1, or with a scrambled siRNA (si-Ctrl) (RiboBio, Guangzhou, China), by using HiPerFect Transfection Reagent (#301705, Qiagen, Hilden, Germany).

The siRNA sequences are as follows:

RAGEsense: 5′-GGAAUGGAAAGGAGACCAATT-3′

anti-sense: 5′-CCUUACCUUUCCUCUGGUUAA-3′

Caveolin-1sense: 5ʹ-CGAGAAGCAAGUGUACGACdTdT-3ʹ.

anti-sense: 5ʹ-GUCGUACACUUGCUUCUCGdTdT-3ʹ.

NF-κB p65sense: 5′-GGAGUACCCUGAGGCUAUAACUCGC-3′

anti-sense: 5′-GCGAGUUAUAGCCUCAGGGUACUCCAU-3′

### Western blotting

HUVECs pre-treated as indicated were lysed with ice-cold RIPA buffer supplemented with protease inhibitor and phosphatase inhibitor cocktails (Beyotime, China). Lysates of equal amounts of total protein were subjected to SDS-PAGE to resolve the proteins, which were subsequently electrotransferred onto PVDF membranes (Millipore, USA). After blocking the membranes with 5% milk for 1 h at room temperature, they were incubated with primary antibodies overnight at 4 °C. Subsequently, they were washed three times in TBST and then incubated with appropriate secondary antibodies (Proteintech, Wuhan, China) for 1 h at room temperature. Enhanced chemiluminescence (Millipore, USA) was used to visualize target proteins, and images were acquired using a bio-imaging system. Densitometric analysis of protein bands was performed using the ImageJ software.

### Immunofluorescence analysis

To visualize the translocation of NF-κB p65 in HUVECs, the cells were plated on confocal dishes and grown to confluence. After the cells were treated as indicated, they were fixed and permeabilized using 4% PFA and 0.5% Triton X-100, respectively. Afterward, the cells were washed twice with PBS, blocked with 5% BSA for 1 h at 37 °C, and then incubated with a primary antibody against NF-κB p65 (1: 50) overnight at 4 °C. Subsequently, the samples were incubated at 37 °C in the dark with an AF488–conjugated secondary antibody for 1 h and then with DAPI for 5 min. Finally, the samples were analyzed using a confocal laser scanning microscope (Olympus, Japan). Images were taken randomly from three fields of view of each biological replicate. The investigator who analyzed the staining was blinded to the treatment group.

### Luciferase reporter assay

The luciferase reporter system was obtained from DesignGene (Shanghai, China). In brief, the Caveolin-1 promoter was inserted into the luciferase reporter vector pGL3-Basic, which carried the SV40 promoter upstream of the firefly luciferase reporter gene. As a systemic control, the pGL3-empty vector was transferred. HUVECs were seeded in 24-well plates and cultured overnight. Subsequently, the cells were co-transfected with 500 ng pcDNA3.1-NF-κB p65 or pcDNA3.1 (mock); 500 ng pGL3-Basic, pGL3-RAGE or pGL3-Caveolin-1; and 100 ng of the renilla luciferase vector pRL-TK (internal control) by using Lipofectamine 2000 (Invitrogen). After 48 h, the cells were harvested, and their firefly luciferase and Renilla luciferase activity was quantitated using a dual-luciferase reporter assay kit (Yeasen, China). The firefly luciferase signal was normalized to the Renilla luciferase signal.

### Analysis of atherosclerosis in mice

All the animal experiments were performed in accordance with the ARRIVE 2.0 guideline. Thirty-six male *ApoE*^−/−^ mice (on C57BL6 /J background, purchased from Beijing HFK Bioscience) were provided with standard chow for 1 week. At 6 weeks of age, the mice were randomly allocated into two groups (n = 18) according to their body weight. Each mouse was injected with rAAV9-ICAM2-sh-Ctrl or rAAV9-ICAM2-sh-RAGE (5 × 10^11^ vg/mouse), which knocks down RAGE specifically in ECs, through the tail vein (Cowan et al. 1998; Cheng et al. 2023). After 4 weeks, the mice in each group were randomized and then treated with either BSA or AGEs (50 μg/mouse/2 weeks, i.v., n = 9 per treatment) under HFD treatment for 12 weeks (Guo et al. 2021). Subsequently, the mice were euthanized under anesthesia. The heart and aorta were isolated and then fixed with 4% PFA for 24 h. The aortic arch was excised for hematoxylin–eosin (HE) staining, and the whole aorta was stained with Oil red O. Additionally, the hearts were placed in the optimal cutting temperature compound (Tissue-Tek) and then frozen at − 80 °C. Starting from the aortic valve, the aortic root was continuously sectioned at 5 μm intervals until all the three leaflets of the aortic valve were visible. Oil Red O was used to stain the sections. In sections where HE staining was performed, cellular-stained and acellular areas in intimal lesions were quantified as the total atherosclerotic lesion area. In samples subjected to Oil red O staining, areas stained red were considered atherosclerotic lesions, and the percentage of atherosclerotic lesion area/total surface area was quantified using Image J software. Photos were taken using a digital camera (Canon EOS 6D2, Tokyo, Japan).

To avoid the analysis bias, the identification of the histological sections was blinded throughout processing. An expert in pathology reviewed all aorta histological mice samples, and representative tissue samples were selected.

### Immunohistochemical analysis

Sections from the aortic tissue of mice were processed as described above and stained with antibodies specific for RAGE (1:2000, 16346-1-AP, Proteintech, Wuhan, China), Caveolin-1 (1:400, #3267, CST, USA), p-p65 (1:50, AP0124, ABclonal, Wuhan, China), NF-κB p65 (1:50, 10745-1-AP, Proteintech, Wuhan, China), and IκBα (1:50, 10268-1-AP, Proteintech, Wuhan, China).

### Statistical analysis

All the experiments were performed in triplicate and are presented as mean ± SEM. Statistical analyses were conducted using the GraphPad Prism 8 Software. Our data passed normality and equal-variance tests. Two groups were compared using unpaired Student's *t*-test, and multiple group comparisons were performed using one-way ANOVA with Tukey post-hoc test. Statistical significance (*) was set at p-value < 0.05.

## Results

### Effect of AGEs on the viability of HUVECs

**Fig. 1:**
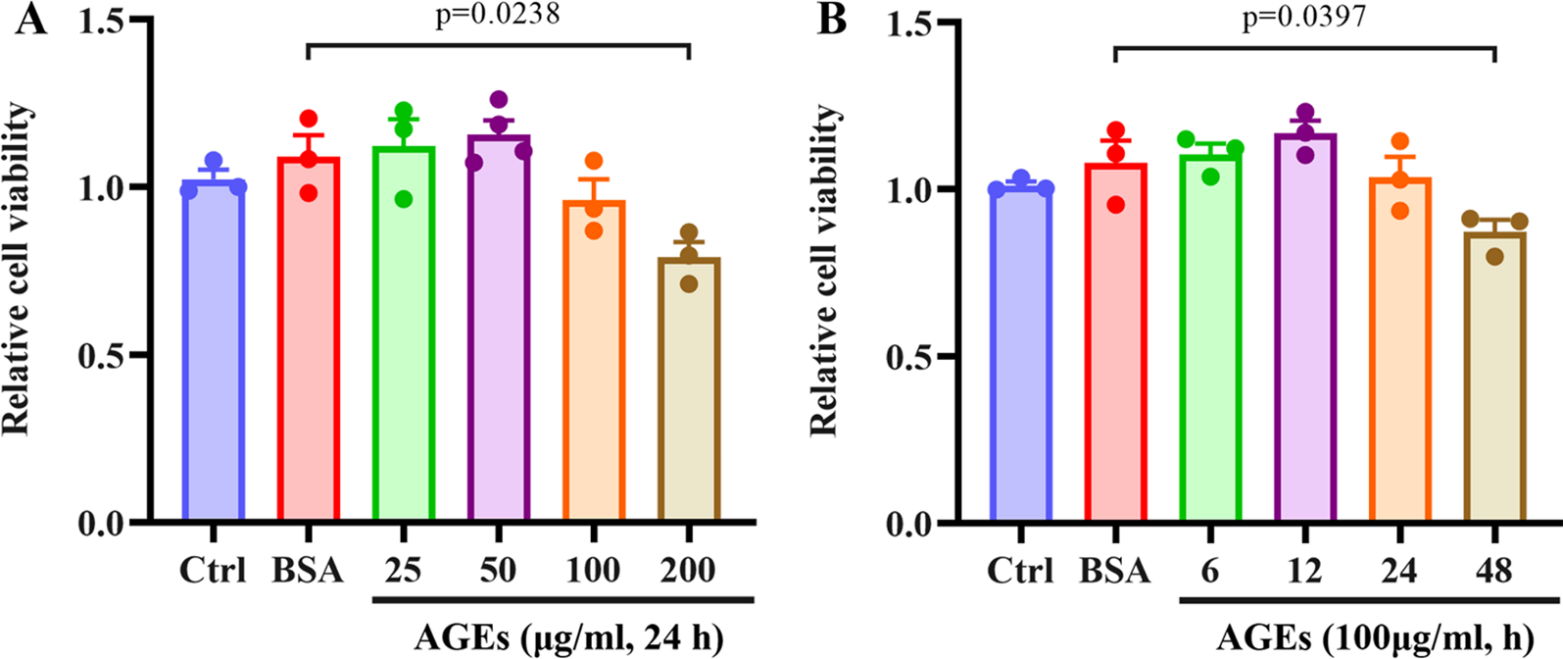
Cell viability in AGEs-treated HUVECs. **A** Cells were treated with various concentrations of AGEs, and cell viability were measured using the CCK-8 assay. **B** The cells were treated with AGEs 100 µg/mL for different time, and the cell viability was determined by CCK-8 assay; n = 3

Determining a non-toxic, yet pathological, concentration was the goal. Dosage optimization with various AGEs concentrations to find an appropriate experimental concentration. AGEs at < 100 µg/mL had no significant cytotoxic effect on HUVECs compared with the BSA-treated group (Fig. 1A**)**. However, the cell viability decreased at 200 μg/mL. Next, we determined the cytotoxicity of 100 µg/mL AGEs at various time points (6, 12, 24, and 48 h). We observed that AGEs had no visible cytotoxic effect on HUVECs for 24 h but had a toxic effect afterward (> 24 h) (Fig. 1B). In addition, compared with the control group, BSA treatment did not change the cell viability, indicating that BSA had no cytotoxicity to HUVECs.

Circulating levels of AGEs in type 2 diabetes with cardiovascular complications generally do not exceed 21.4 µg/ml (Sabbatinelli et al. 2022). The process by which AGEs promote AS involves continuous stimulation at low concentrations in vivo; however, cells cannot endure long-term exogenous AGEs treatment in vitro. Hence, we employed a relatively high concentration of AGEs and a relatively long treatment time (100 µg/mL, 24 h) to mimic the physiological state.

### AGEs promote LDL transcytosis and uptake in HUVECs

**Fig. 2:**
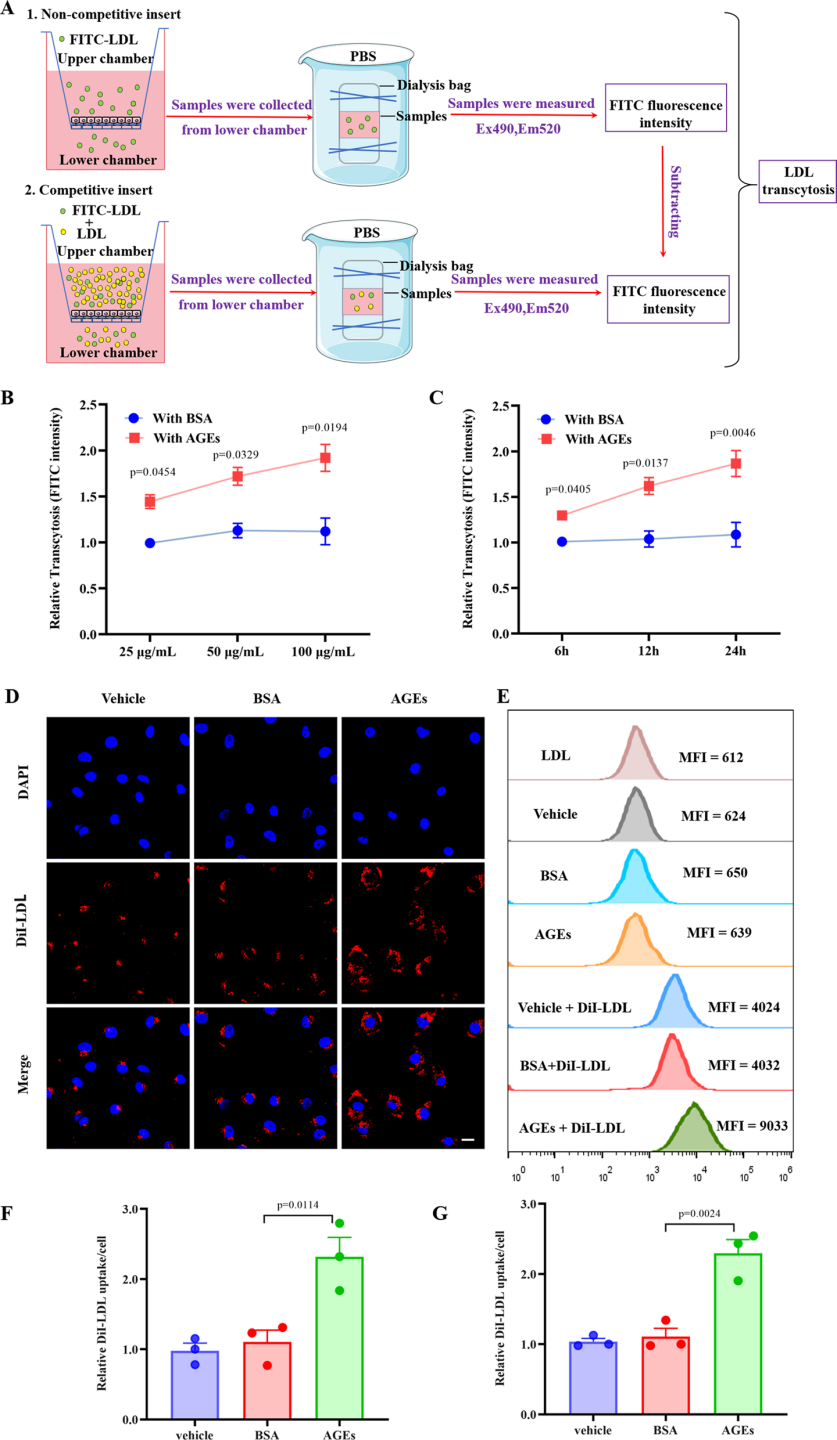
AGEs promote LDL transcytosis and uptake in HUVECs. **A** Schematic diagram of FITC-LDL transcytosis across endothelial cells. **B** LDL transcytosis in HUVECs under different concentrations (25 µg/mL, 50 µg/mL, 100 µg/mL) of BSA or AGEs treatment; n = 3. **C** LDL transcytosis in HUVECs under different times (6 h, 12 h, 24 h) of BSA or AGEs treatmen; n = 3. **D**, **E** HUVECs were treated with vehicle, BSA or AGEs (100 μg/mL, 24 h), followed by incubation with 50 μg/mL DiI-LDL. **D** Representative confocal fluorescent images showing DiI-LDL uptake in HUVECs. The red signals denote DiI-LDL puncta, and the blue DAPI signals represent nuclei (*n* = 3). Scale bars: 20 μm. **E** Cells were harvested, and the MFI of DiI-LDL in cells was measured by flow cytometry analyses. **F** Quantification of DiI-LDL uptake by HUVECs; n = 3. **G** Quantification summary of DiI-LDL uptake represented by MFI in HUVECs; n = 3

To determine whether AGEs can modulate LDL transcytosis across HUVECs, we established a transcytosis model utilizing the excess-LDL competition approach (Bian et al. 2014) (Fig. 2A). AGE treatment significantly facilitated the LDL transcytosis across a monolayer of HUVECs in dose- and time-dependent manners (Fig. 2B, C). In contrast, BSA had no effect.

Since EC uptake of LDL is an intermediary stage in LDL transcytosis, cellular LDL level can be utilized as an indicator of LDL transcytosis (Bai et al. 2020a, 2020b). Accordingly, we calculated the fluorescent intensity of each cell via confocal imaging and flow cytometry to quantify the cellular DiI-LDL uptake. We observed that HUVECs incubated with DiI-LDL contained small and distinct fluorescent vesicles (Fig. 2D). AGE treatment enhanced the DiI-LDL fluorescence intensity in HUVECs, indicating increased LDL uptake (Fig. 2D, F). Likewise, the MFI of DiI-LDL in HUVECs, which represents the level of DiI-LDL uptake, was significantly elevated in cells treated with AGEs (Fig. 2E, G). As expected, DiI-LDL uptake was not substantially different in the BSA-treated group, implying that BSA did not affect the LDL uptake in HUVECs. In conclusion, AGEs can promote LDL uptake and transcytosis in HUVECs.

### Caveolin-1 plays an important role in AGE-induced LDL transcytosis

**Fig. 3:**
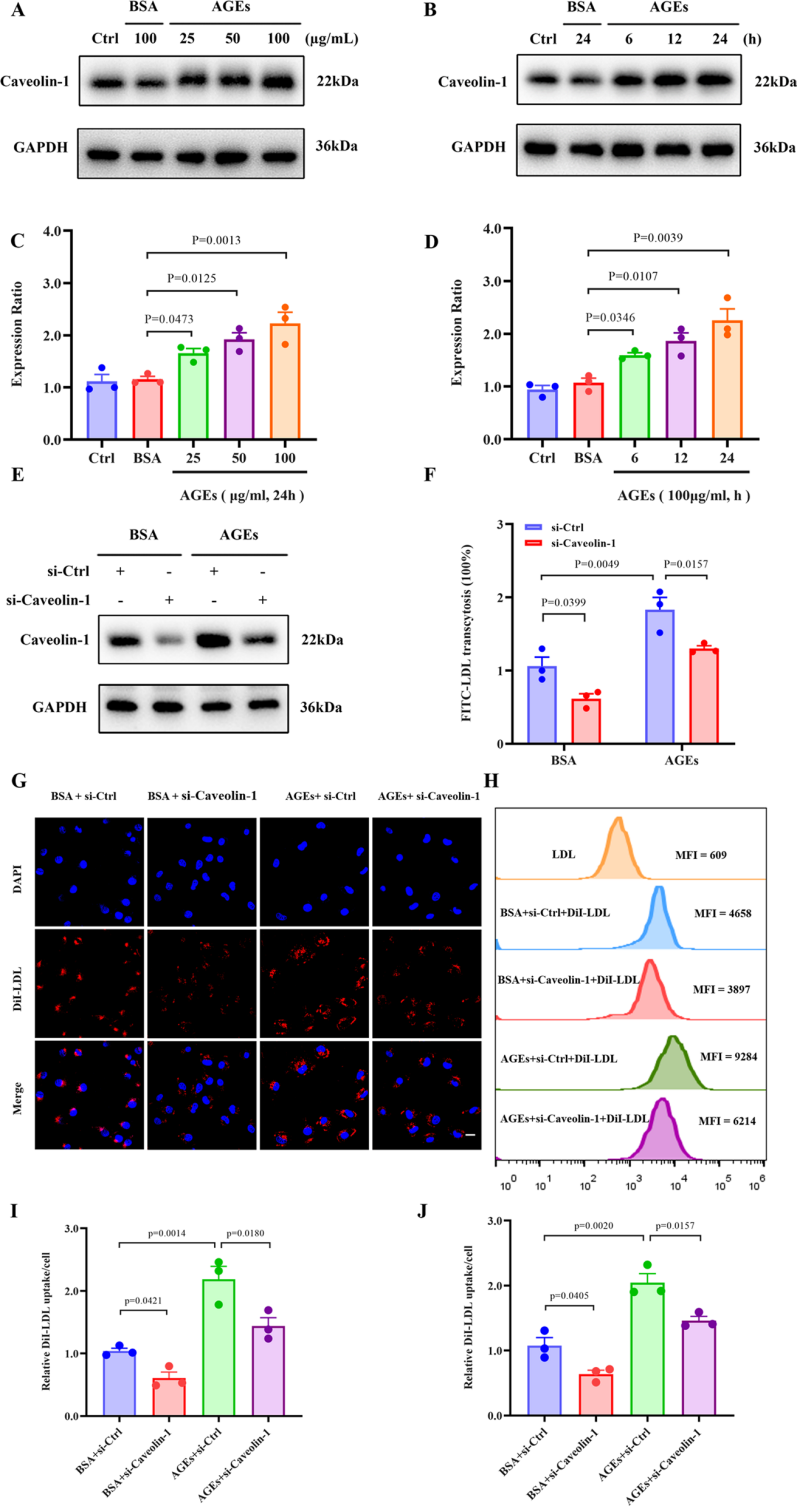
Caveolin-1 plays an important role in AGEs-mediated LDL transcytosis. **A**, **B** Dose–response (left top) and time course (right top) of Caveolin-1 protein induction by AGEs. **C**, **D** Summary bar graphs showing the expression of the indicated proteins; n = 3. **E**, **F** Cells were transfected with either control or Caveolin-1 siRNA for 48 h and then treated with AGEs for 24 h. **E** Western blots are shown for Caveolin-1. F Normalized amount of LDL transcytosis; n = 3. **G**–**H** HUVECs were transfected with either control or Caveolin-1 siRNA for 48 h and then treated with AGEs for 24 h, followed by incubation with 50 μg/mL DiI-LDL. **G** Representative confocal fluorescent images showing DiI-LDL uptake in HUVECs. The red signals denote DiI-LDL puncta, and the blue DAPI signals represent nuclei. Scale bars: 20 μm. **H** Cells were harvested, and the MFI of DiI-LDL in cells was measured by flow cytometry analyses. **I** Quantification of DiI-LDL uptake by HUVECs; n = 3. **J** Quantification summary of DiI-LDL uptake represented by MFI in HUVECs; n = 3

Caveolin-1, a plasma-membrane protein implicated in caveolae formation, is essential for LDL transcytosis in ECs and thereby promotes AS (Ramírez et al. 2019). Additionally, Caveolin-1 deficiency can significantly inhibit the formation of atherosclerotic plaques (Frank et al. 2004; Fernández-Hernando et al. 2009). Given the essential role of Caveolin-1 in LDL transcytosis, we postulated that AGEs promote LDL transcytosis through this protein. As indicated in Fig. 3A–D) AGEs could augment the expression of Caveolin-1 in concentration- and time-dependent manners. In contrast, BSA had no significant effect on the expression of Caveolin-1. Furthermore, the AGE-induced LDL uptake and transcytosis in HUVECs were significantly downregulated by an siRNA targeting Caveolin-1 (Fig. 3E–J). The above results suggest that AGEs can significantly upregulate Caveolin-1, which in turn boosts LDL transcytosis across ECs.

### RAGE is involved in AGE-induced, Caveolin-1–mediated LDL uptake

**Fig. 4:**
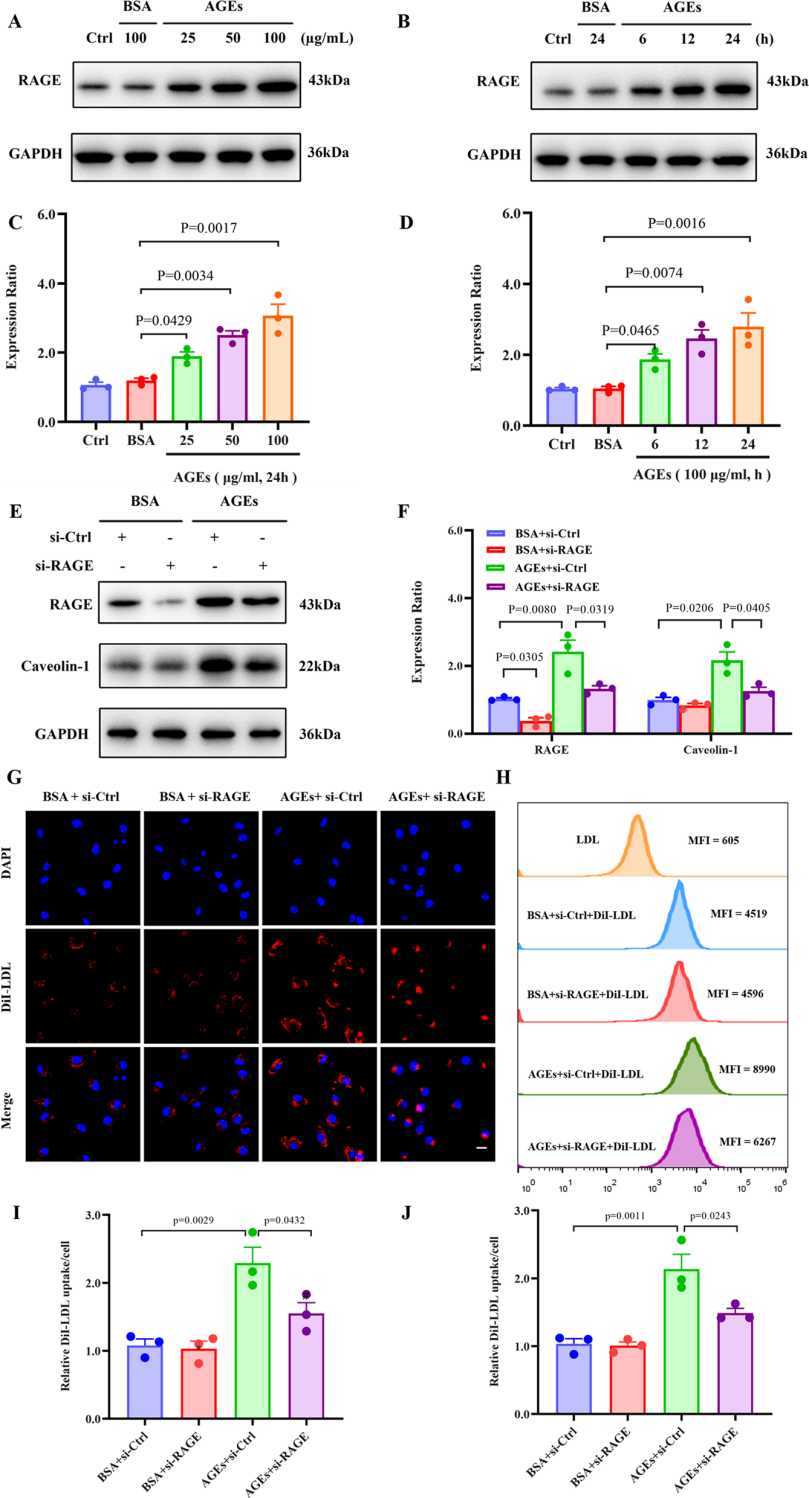
RAGE is involved in AGEs-regulated Caveolin-1 mediated LDL uptake. **A**, **B** Dose–response (left top) and time course (right top) of RAGE protein induction by AGEs. **C**, **D** Summary bar graphs showing the expression of the indicated proteins; n = 3. **E**, **F** Cells were transfected with either control or RAGE siRNA for 48 h and then treated with AGEs for 24 h. Representative western blotting analyses of the indicated proteins **E** and summary bar graph showing the expression of the indicated proteins (**F**). **G**–**H** HUVECs were transfected with either control or RAGE siRNA for 48 h and then treated with AGEs for 24 h, followed by incubation with 50 μg/mL DiI-LDL. **G** Representative confocal fluorescent images showing DiI-LDL uptake in HUVECs. The red signals denote DiI-LDL puncta, and the blue DAPI signals represent nuclei. Scale bars: 20 μm. **H** Cells were harvested, and the MFI of DiI-LDL in cells was measured by flow cytometry analyses. **I** Quantification of DiI-LDL uptake by HUVECs (n = 3). **J** Quantification summary of DiI-LDL uptake represented by MFI in HUVECs (n = 3)

Considering that RAGE is the most important receptor for AGEs and appears to be localized to caveolae within ECs (Lisanti et al. 1994), we assessed for an association between RAGE and Caveolin-1 expression. Indeed, AGEs upregulated RAGE in HUVECs in dose- and time-dependent manners (Fig. 4A–D). Moreover, we found that knocking down RAGE downregulated Caveolin-1 in HUVECs (Fig. 4E, F). In addition, the RAGE knockdown substantially attenuated the DiI-LDL fluorescence intensity in the cells (Fig. 4G–J), thus implying reduced LDL uptake. Together, these results suggest that RAGE functions upstream of Caveolin-1 and likely modulates Caveolin-1 expression in AGE-treated HUVECs.

### AGEs promote Caveolin-1–mediated LDL transcytosis through a positive feedback loop between RAGE activity and the NF-κB signaling

**Fig. 5:**
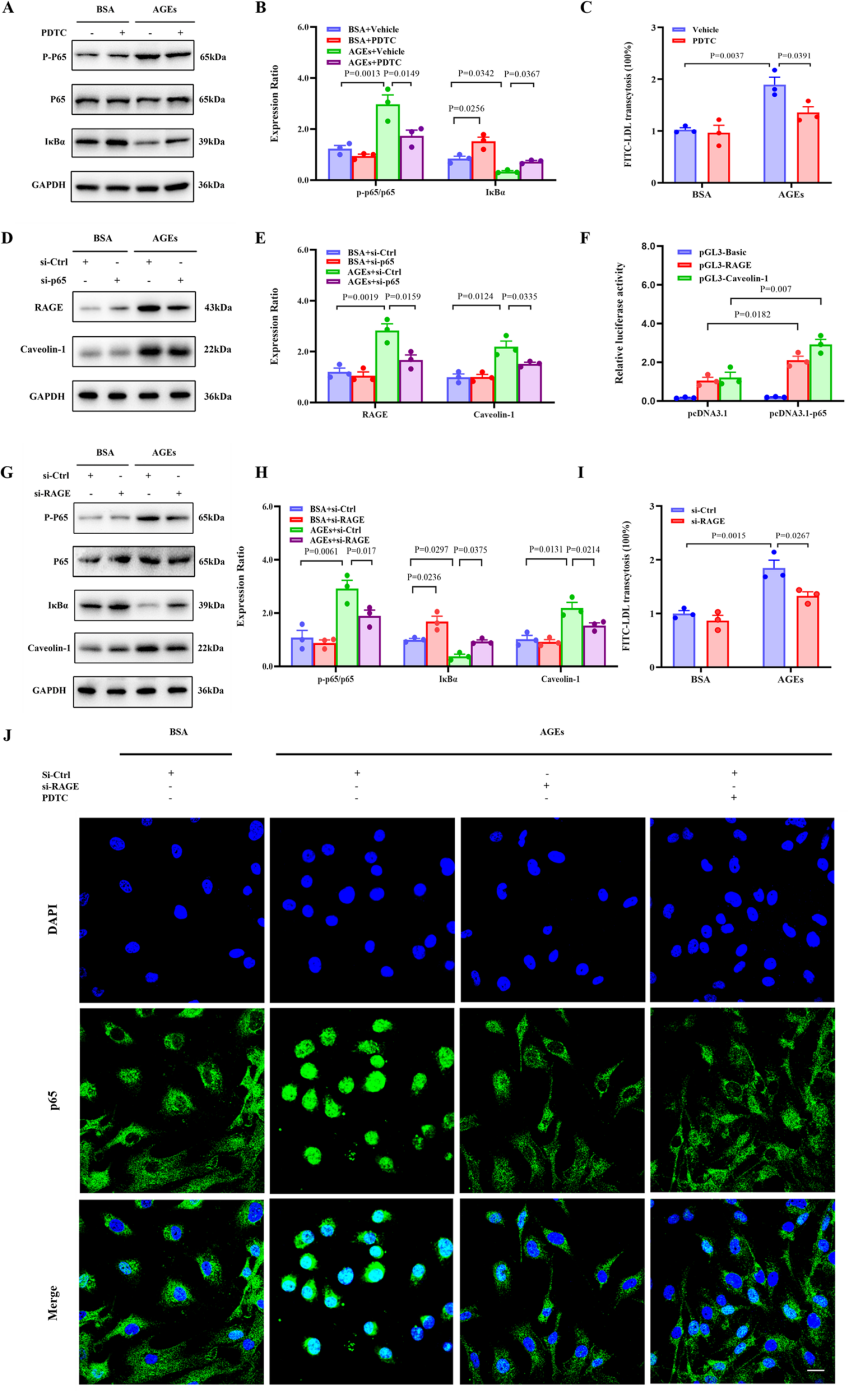
AGEs promote Caveolin1-mediated LDL transcytosis through the NF-κB-RAGE-NF-κB signaling loop. **A**–**C** After pretreating HUVECs with PDTC (10 μmol/ L) for 1 h, HUVECs were exposed to BSA or AGEs for 24 h. Western blot was used to detect the expression of NF-κB related molecules **A** and summary bar graph showing the expression of the indicated proteins (**B**). Normalized amount of LDL transcytosis(**C**); n = 3. **D**–**G** 48 h after transfection with control or NF-κB p65 siRNA, HUVECs were treated with BSA or AGEs for 24 h, and the expression of RAGE and Caveolin-1 in HUVECs were detected by Western blot. Representative western blotting analyses of the indicated proteins **D** and summary bar graph showing the expression of the indicated proteins (**E**); n = 3. **F** Dual luciferase reporter assay was performed to measure the activities of the RAGE promoter or Caveolin-1 promoter when PGL3-RAGE or PGL3-Caveolin-1 co-infiltrated with or without pcDNA3.1-NF-κB p65 in HUVECs; n = 3. **G**–**I** 48 h after transfection with control or RAGE siRNA, HUVECs were treated with BSA or AGEs for 24 h, the expression of NF-κB related molecules and Caveolin-1 in HUVECs were detected by Western blot **G** and summary graph showing the expression of the indicated proteins (**H**). Normalized amount of LDL transcytosis (**I**); n = 3. **J** Representative immunofluorescence images of p65 in in HUVECs treated as above. Scale bar, 20 mm

AGEs increase inflammation by binding to RAGE (Uribarri et al. 2007; Volz et al. 2012). One of the earliest events following RAGE activation is the activation of the transcription factor NF-κB (Anisuzzaman et al. 2014), which generally involves degradation of IκBα subunit, phosphorylation of the p65 subunit and translocation into the nucleus. Consistent with this notion, we observed that the NF-κB signaling pathway was activated by AGEs, and this effect was downregulated by PDTC, a specific inhibitor of NF-κB that suppresses the degradation of the inhibitory subunit IκBα (Fig. 5A, B). Moreover, the inhibition of NF-κB reduced the AGE-induced FITC-LDL transcytosis in vascular ECs (Fig. 5C). To further clarify the contribution of the NF-κB signaling to AGE-induced LDL transcytosis, we transfected HUVECs with an siRNA against NF-κB p65 for 48 h and then measured the effect of the knockdown on the expression of RAGE and Caveolin-1, which are related to AGE-induced LDL transcytosis. Surprisingly, both RAGE and Caveolin-1 were upregulated by NF-κB p65, and these effects were reversed by p65 siRNA (Fig. 5D, E). Next, a dual-luciferase reporter assay was used to assess whether NF-κB can bind to the RAGE and Caveolin-1 promoters. NF-κB p65 overexpression significantly increased the luciferase activity of the reporter in HUVECs (Fig. 5F). These results showed that the RAGE and Caveolin-1 promoters contain sites targeted by NF-κB p65, and the activation of the NF-κB signaling pathway promotes the expression of RAGE and Caveolin-1.

Interestingly, in AGE-treated HUVECs, knocking down RAGE impeded the phosphorylation of p65 and upregulated IκBα (Fig. 5G, H), and both the RAGE knockdown and PDTC treatment attenuated the AGE-induced nuclear translocation of p65 in HUVECs (Fig. 5J). These data suggest a positive feedback loop between the NF-κB signaling and RAGE expression, and inhibition of RAGE demotes the activation of the NF-κB signaling pathway to some extent. Furthermore, RAGE inhibition downregulates Caveolin1 level and reduces LDL transcytosis in AGE-treated HUVECs (Fig. 5G and I).

Taken together, in HUVECs, RAGE inhibition suppresses the AGE-induced activation of the NF-κB signaling, consequently inhibiting the increase in Caveolin-1 level and ameliorating LDL transcytosis.

### Knocking down RAGE suppresses diabetic AS in mice

**Fig. 6:**
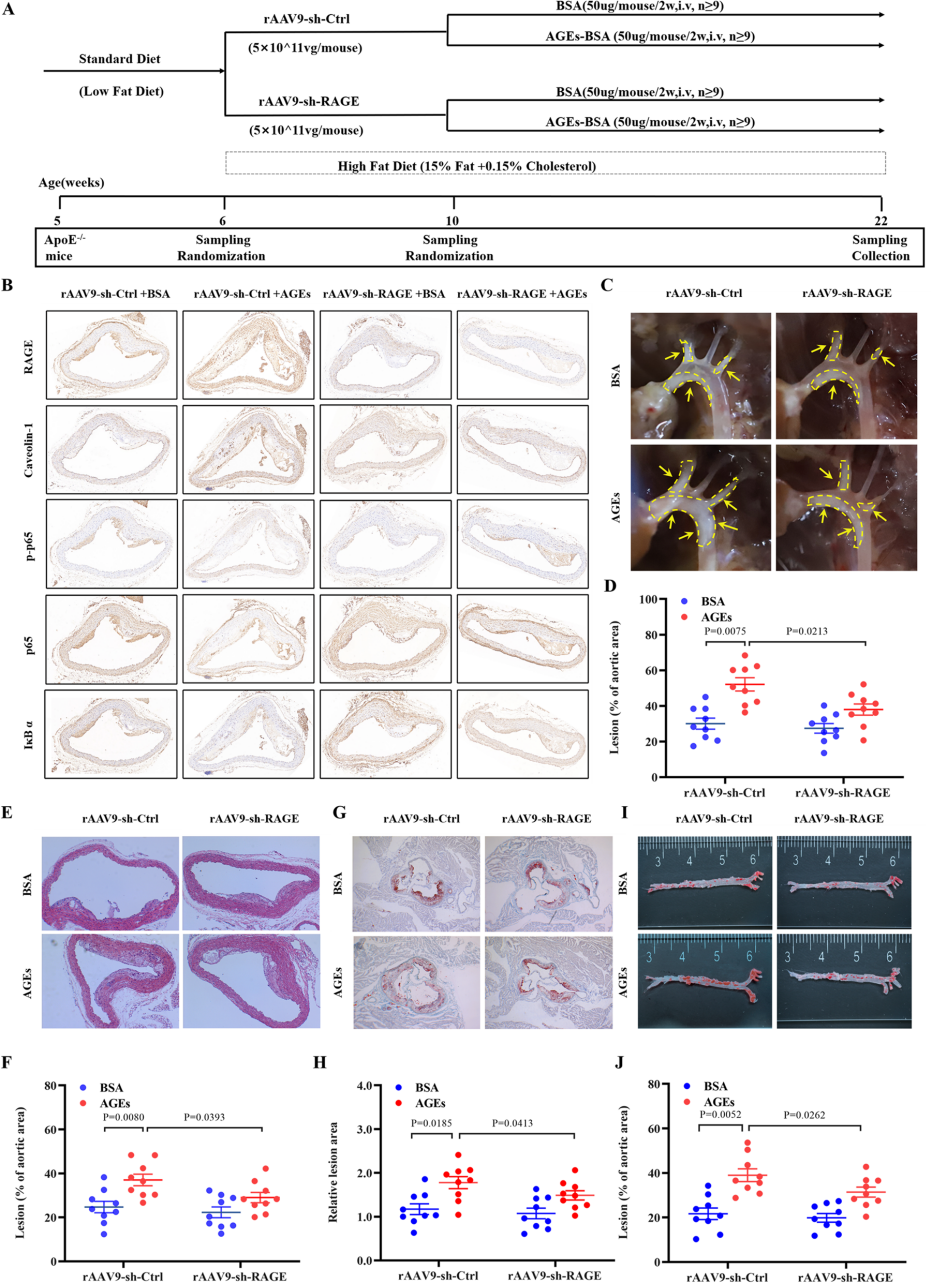
RAGE silencing alleviated diabetic AS in *ApoE*^−/−^ mice*.* **A** Schematic diagram of the animal experimental process. **B** The representative images of immunohistochemistry (IHC) staining for RAGE, Caveolin-1, phosphorylated p65 (p-p65), NF-κB (p65) and IκBα protein expression. **C** Representative micro pictures of the aortic arches from *ApoE*^−/−^ mice. Arrows and dashed lines indicate atherosclerotic plaques in the brachiocephalic artery, subclavian artery and lesser curvature of the aortic arch. **D** The percentage of aortic arch lesion area in the indicated group (n = 9). **E**, **F** Representative histological analysis of brachiocephalic arteries isolated from *ApoE*^−/−^ mice and stained with HE and the percentage of positively regions in the indicated groups (n = 9). **G**, **I** Representative images of Oilred O-stained aortic root sections and the lesion area in the indicated groups (*n* = 9). Scale bars: 500 μm. **H**, **J** Representative images of aorta sections with Oil Red O-stained aortas (en face) and the percentage of positively regions in the indicated groups (*n* = 9)

Next, we used a viral system to knock down RAGE in the endothelium of a mouse model of AS and thereby observed the effect of RAGE deficiency on atherogenic progression in vivo. Adeno-associated viruses (AAVs) constructed for EC-specific expression of an shRNA against RAGE (rAAV9-sh-RAGE) or those expressing a control shRNA (rAAV9-sh-Ctrl) were injected into *ApoE*^−/−^ mice through the tail vein (Fig. 6A). After 4 weeks, the mice were administered BSA or AGEs (50 µg/mouse/2 weeks) for 3 months. Finally, the mice were sacrificed, and their hearts and aortas were histologically examined. Immunohistochemistry was used to examine the expression of Caveolin-1 and NF-κB in the mouse aortic tissues derived from AS mice. The results showed an increase in the expression of RAGE, Caveolin-1, and P-P65, in the aorta of AGEs-treated AS mice, while the expression of IκBα was significantly decreased (Fig. 6B). However, these effects were reversed by rAAV9-sh-RAGE, indicating inhibition of the RAGE/NF-κB/Caveolin-1 Pathway.

Furthermore, the AGEs group had significantly larger atherosclerotic lesions compared with the BSA group (Fig. 6C). HE staining and Oil Red O staining also indicated increased lipid deposition in the sub-endothelial space of the AGEs group, compared with the BSA group (Fig. 6E–J). However, rAAV9-sh-RAGE suppressed these effects, implying that the RAGE knockdown suppressed the AGE-induced AS. All these results suggest that RAGE intervention delays the pathogenesis of AGE-induced atherosclerotic lesions.

## Discussion

**Fig. 7:**
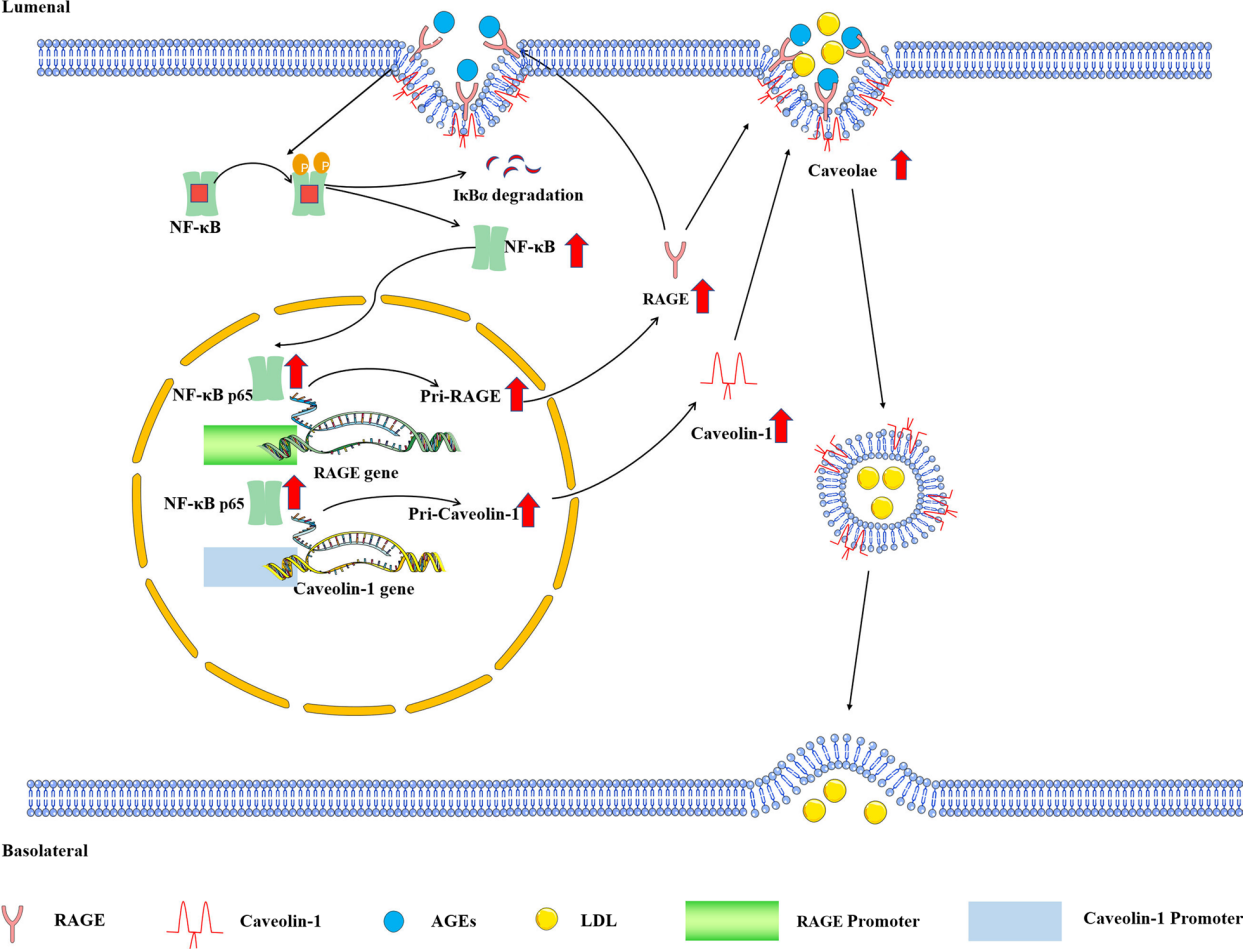
The mechanism of RAGE/NF-κB/Caveolin-1 pathway in AGEs-stimulated LDL transcytosis. Working model: the activation of the RAGE/NF-κB/Caveolin-1 axis plays a vital role in AGEs-promoted LDL transcytosis across ECs and development of AS. when exposed to AGEs, the RAGE-mediated NF-κB signaling is activated, which induces the expression of Caveolin-1, a main coat protein of caveolae and involved in LDL transcytosis in ECs. The downregulation of RAGE blocks NF-κB-RAGE-NF-κB signaling loop, resulting in a drop in Caveolin-1 expression and less LDL transcytosis, which ultimately delaying the development of AS. *NF-κB* nuclear factor kappa B, *ECs* endothelial cells, *mRNA* messenger ribonucleic acid, *pri-RAGE* primary transcript of RAGE, *pri-Caveolin-1* primary transcript of Caveolin-1

In this study, we demonstrated that AGEs promote lipid transcytosis in the vascular endothelium and thereby accelerate the formation of atherosclerotic lesions. AGEs promote Caveolin-1–mediated LDL uptake and transcytosis by interacting with their plasma-membrane–localized receptor RAGE. Activation of RAGE by AGEs induces the NF-κB signaling pathway, which upregulates RAGE in a positive feedback loop that ultimately leads to a pathophysiological cascade. The schematic diagram of our study is shown in Fig. 7.

AS is the main cause of coronary heart disease, cerebral infarction, and peripheral vascular disease. A growing body of evidence suggests that retention of apoB-containing lipids, such as LDL, is the fundamental event leading to sub-endothelial accumulation of lipids and development of AS (Tabas et al. 2007). This notion is commonly referred to as the "response to retention hypothesis," which is the initiating event in the pathogenesis of AS (Fogelstrand and Borén 2012).

AGEs are heterogeneous compounds that primarily emerge from the non-enzymatic interaction of macromolecules with glucose. Cellular interactions with AGEs cause physiological reactions that have been related directly to the occurrence of DM-related diseases and are mediated by certain cell-surface receptors (Zhu et al. 2012). AGEs are closely associated with diabetic AS (Kopytek et al. 2020); however, the specific molecular mechanism whereby AGEs promote AS is not well understood even though abnormal lipid metabolism is known to be the pathological basis of AS (Gordts et al. 2014). In the presented study, we hypothesized that AGEs are involved in LDL transcytosis, and to test this hypothesis, we established an *in-vitro* transcytosis model using HUVECs. Indeed, we observed that AGEs induced LDL transcytosis in HUVECs.

Currently, caveolae are thought to play a crucial role in LDL transcytosis, and we observed that knocking down Caveolin-1 effectively inhibited AGE-induced LDL uptake and transcytosis in HUVECs.

RAGE is a pattern recognition receptor capable of binding a wide variety of AGEs and non-AGE ligands, such as Mac-1, s100/calgranulins, HMGB1, and amyloid fibers. These ligands share various structural features; for example, RAGE recognizes its ligands via their multiple β-sheets. RAGE expression is focally enhanced in atherosclerotic vascular disease, paralleling the aggregation of RAGE ligands (Arumugam et al. 2012). Following the binding of its ligand, RAGE initiates a signaling cascade through activation of the transcription factor NF-kB, and oxidative stress and inflammatory responses. RAGE mediates a signaling cascade that results in its own upregulation and a persistent inflammatory state through a positive feedback loop (Wang et al. 2009). Our findings suggest that RAGE and Caveolin-1 are upregulated by AGEs, and knocking down RAGE inhibits AGE-induced upregulation of Caveolin-1, implying a correlation between RAGE and Caveolin-1. Therefore, RAGE may modulate cellular Caveolin-1 level in HUVECs under diabetic conditions.

Furthermore, previous studies by our group have shown that NF-κB can upregulate Caveolin-1 (Zhang et al. 2014). In this study, the Caveolin-1 expression in AGE-treated HUVECs was increased through the NF-κB activity. Therefore, we hypothesized that RAGE upregulated Caveolin-1 in HUVECs through the NF-κB signaling. The results from our dual-luciferase reporter assay demonstrated that NF-κB p65 can bind to the Caveolin-1 promoter. Together, our results suggest that activation of the NF-κB signaling pathway increases Caveolin-1 expression and induces LDL transcytosis in ECs, which ultimately promotes the pathogenesis of AS. The recruitment of p65 to these promoters was eliminated in cells transfected with an siRNA targeting p65, and the LDL transcytosis was consequently decreased. Importantly, our data suggest that the RAGE-dependent NF-kB activation upregulates not only Caveolin-1 but also RAGE, and this positive feedback loop eventually results in a pathophysiological cascade. Inhibiting the RAGE activity suppresses this feedback in AGE-treated HUVECs, thereby suppressing the RAGE-NF-κB-Caveolin-1 cascade and LDL transcytosis.

It has been reported that hyperglycemia may promote the formation of AGEs, and many investigations have exclusively focused on the function of hyperglycemia in DM, ignoring the effects of AGEs. To probe the role of endothelial AGEs/RAGE in AS in vivo, we used an AS model utilizing HFD-fed *ApoE*^−/−^ mice. A considerable increase in atherosclerotic plaque area was found in *ApoE*^−/−^ mice upon administration of AGEs, but this effect was suppressed when the mice were pre-treated with rAAV9-sh-RAGE. These data suggest that knocking down RAGE significantly suppresses the AGE-induced LDL transcytosis across ECs in AS mice, as indicated by smaller plaques.

There are some limitations to this study. Firstly, not enough vascular samples from patients were collected to further enrich our experiments. Secondly, soluble RAGE (sRAGE) lacks transmembrane structural domains and intracytoplasmic structural domains (Koyama et al. 2007). As a natural antagonist, it antagonizes RAGE signaling in vitro and in vivo by binding to ligands such as AGEs (Leung et al. 2022). Thus, sRAGE could serve as an alternative to RAGE siRNA; however, only the latter was chosen for our study. Finally, the elevation of Caveolin-1 may be due to its increased synthesis and decreased protein degradation, whereas ubiquitination degradation and autophagic degradation of Caveolin-1 were not investigated in the present study, which will be the focus of our future research.

## Conclusions

Taken together, this study demonstrated that the AGE-induced RAGE/NF-κB axis can upregulate Caveolin-1, which increases the sub-endothelial deposition of LDL and ultimately the formation of atherosclerotic plaques. From the perspective of LDL transcytosis across ECs, we present a novel mechanism underlying the pathogenesis of diabetic AS and propose RAGE as a potential therapeutic target in DM-related cardiovascular disease.

## Data Availability

The datasets used and/or analyzed during the current study are available from the corresponding author on reasonable request.

## Abbreviations

AGEs: Advanced glycation end products
RAGE: Receptor for advanced glycation end products
CCK-8: Cell Counting Kit-8
HUVECs: Human umbilical vein endothelial cells
LDL: Low density lipoprotein
AS: Atherosclerosis
siRNA: Small interfering RNA
H&E: Hematoxylin and eosin
